# Repeat ultrasound in pediatric EDs improves appendicitis diagnosis after referral imaging

**DOI:** 10.3389/fped.2025.1676690

**Published:** 2025-10-28

**Authors:** Vitaliy Perepelitsa, Jeffrey Ames, Rahul Kaila, Nicholas Sausen, Kari Schneider, Scott Lunos, Bradley Segura, Pablo Avendano, Jeffrey P. Louie

**Affiliations:** 1 Department of Pediatrics, University of Minnesota, Minneapolis, MN, United States; 2 Department of Radiology, University of Minnesota, Minneapolis, MN, United States; 3 Clinical and Translational Science Institute, University of Minnesota, Minneapolis, MN, United States; 4 Department of Surgery, University of Minnesota, Minneapolis, MN, United States

**Keywords:** ultrasound, pediatric emergency, appendicitis, diagnostic accuracy, referring emergency department

## Abstract

**Background:**

Acute appendicitis is a leading cause of surgical emergencies in children, with ultrasound (US) emerging as a preferred diagnostic tool due to its lack of radiation and cost-effectiveness. However, the accuracy of US is highly operator-dependent and may vary between general referring emergency departments (EDs) and specialized pediatric EDs.

**Objective:**

To compare the diagnostic sensitivity and specificity of US performed at referring EDs vs. a pediatric ED in identifying acute appendicitis.

**Methods:**

A retrospective study analyzed pediatric patients aged <18 years who underwent US at referring EDs and were transferred to a pediatric ED for repeat imaging between July 2018 and July 2023. Data collected included US findings, surgical pathology, white blood cell count, and patient disposition. Sensitivities of the US were calculated and compared between settings.

**Results:**

Among 64 children included, the US at the pediatric ED demonstrated higher sensitivity (85.2%) compared to referring EDs (51.9%) (*p* = 0.018). Pediatric ED US resulted in fewer non-visualized appendices (a 34.4% reduction) and equivocal findings (a 30.5% reduction). Patients with positive surgical pathology exhibited higher white blood cell counts (mean 17.1) and neutrophil percentages (mean 81.0%). False positive rates were low (6.9%), aligning with published benchmarks.

**Conclusion:**

US performed at pediatric EDs exhibited superior diagnostic accuracy for appendicitis compared to referring EDs, likely due to operator expertise and enhanced imaging protocols. Efforts to standardize training and improve resources at referring EDs may reduce diagnostic disparities and unnecessary interventions.

## Introduction

Acute appendicitis is the most common cause of acute abdominal pain requiring surgery in children and remains a frequent reason for imaging evaluation. Physical findings alone are often sufficient for diagnosis in some cases, but imaging is often obtained to increase diagnostic certainty and guide management (1, 2).

In children, ultrasound is the preferred first-line imaging modality, recommended by the American College of Radiology and other professional guidelines (3–6). Unlike computed tomography (CT), which has high sensitivity but exposes children to ionizing radiation with associated cancer risk (7–9) ultrasound avoids radiation, is cost-effective, and can achieve excellent diagnostic accuracy in experienced pediatric centers (10–12).

Pediatric hospitals report higher rates of appendix visualization and fewer nondiagnostic studies, primarily due to the use of specialized pediatric sonographers, expertise from pediatric radiologists, and the application of standardized techniques such as graded compression and the recognition of secondary signs of appendicitis (13–15).

In contrast, community or referring emergency departments often lack this specialized expertise, and ultrasounds performed in these settings are more likely to be equivocal or inaccurate (16). This can lead to unnecessary CT scans, delays in diagnosis, or inappropriate management (17).

The purpose of this study was to evaluate the accuracy of ultrasounds performed at referring EDs compared with repeat ultrasounds obtained at a dedicated pediatric ED. This was a retrospective case series explicitly designed to highlight the potential inaccuracy of outside ED ultrasounds, and to demonstrate that repeating ultrasound at the pediatric center improves diagnostic accuracy.

## Methods

We conducted a retrospective analysis across a single integrated health system over a five-year period (July 2018–July 2023). Children under 18 years who underwent an ultrasound at a referring ED and then had a repeat ultrasound at the pediatric ED within 12 hours were eligible. Children who underwent an ultrasound only at the outside emergency department (without transfer or repeat ultrasound) were excluded. Paired studies were required to allow for within-patient comparison across settings.

A pediatric radiologist re-evaluated each appendix ultrasound from the referring ED and compared these with the repeat ultrasound performed at the pediatric ED. Each ultrasound was assigned a standardized 4-point score to categorize diagnostic likelihood: (1) appendix visualized and normal; (2) appendix not visualized with no secondary findings; (3) appendix visualized with indeterminate findings or not visualized but secondary findings present; (4) appendix visualized with findings consistent with acute appendicitis. This scoring system was applied uniformly to ultrasounds performed at both referring EDs and the pediatric ED, allowing for direct comparison of diagnostic performance.

Descriptive statistics (means, standard deviations, ranges for continuous variables; counts and percentages for categorical variables) were used to summarize the data collected. Referral and pediatric US results were compared and sensitivity was calculated. SAS V9.4 (SAS Institute Inc., Cary, NC) was used for the analysis. McNemar's test was used to evaluate the statistical significance of differences in diagnostic performance (sensitivity) between US facilities. The study was found exempt by the institutional review board.

## Results

A total of 64 pediatric patients met the inclusion criteria. The median age was 8 years [interquartile range (IQR), 6–10 years], and 58% (*n* = 37) of the participants were male.

### Ultrasound findings

Ultrasound findings were very different between the pediatric ED and the referring EDs (Table 1). When classified using the 4-point ultrasound scoring system:

- Score 1 (normal appendix visualized): 31.3% (20/64) at the pediatric ED vs. 9.4% (6/64) at referring EDs.
- Score 2 (appendix not visualized, no secondary findings): 10.9% (7/64) vs. 45.3% (29/64).
- Score 3 (indeterminate or secondary findings): 20.3% (13/64) vs. 21.9% (14/64).
- Score 4 (findings consistent with appendicitis): 37.5% (24/64) vs. 23.4% (15/64).

**Table 1:** Demographics, ultrasound findings, and clinical outcomes in pediatric appendicitis referrals.

Demographics		
Number of patients	64	
Gender		
Male	37 (57.8%)	
Female	27 (42.2%)	
Median age	8 years old	
Sensitivity results		
Pediatric emergency Department	85.2% (23/27)	
Referral emergency Department	51.9% (14/27)	
Ultrasound results	Pediatric ED	Referring ED
The appendix is visualized and normal.	31.3% (*n* = 20/64)	9.4% (*n* = 6/64)
The appendix is not visualized. There are no findings to support a diagnosis of appendicitis.	10.9% (*n* = 7/64)	45.3% (*n* = 29/64)
The appendix is visualized with an intermediate likelihood of appendicitis, OR The appendix is not visualized, but there are secondary findings present that could be associated with acute appendicitis.	20.3% (*n* = 13/64)	21.9% (*n* = 14/64)
The appendix is visualized with findings consistent with acute appendicitis.	37.5% (*n* = 24/64)	23.4% (*n* = 15/64)
Disposition from ED		
To OR (from ED or after admit)	43.8% (*n* = 28/64)	
Admit then home (no OR)	28.1% (*n* = 18/64)	
Home from ED	28.1% (*n* = 18/64)	
Surgical Pathology results		
Consistent with Appy	93.1% (*n* = 27/29)	
Path not consistent with Appy	6.9% (*n* = 2/29)	

### Diagnostic sensitivity

Using surgical pathology as the reference standard, the sensitivity of ultrasound for appendicitis was significantly higher at the pediatric ED (85.2%, 23/27; 95% CI 66.3%–95.8%) than at the referring EDs (51.9%, 14/27; 95% CI 32.0%–71.3%). This difference was statistically significant (McNemar's test, *p* = 0.0027).

### Disposition and surgical pathology

Of the 64 patients, 28 (43.8%) were taken directly to the operating room. An additional 18 patients (28.1%) were admitted and later discharged without surgery, while the remaining 18 (28.1%) were discharged directly from the ED. Among the 29 patients who underwent surgery, 27 (93.1%) had confirmed appendicitis on pathology. Two patients (6.9%) had normal appendices and were both classified as “intermediate” on ultrasound.

### Laboratory findings

Patients with pathology-confirmed appendicitis had significantly higher mean white blood cell counts (17.1, SD 6.3) and neutrophil percentages (81.0%, SD 9.0) compared to those without appendicitis, who had a mean WBC of 11.1 (SD 5.4) and neutrophils of 69.2% (SD 18.2), as shown in Table 2.

**Table 2:** Detailed clinical and pathology findings in negative appendicitis cases.

	Patient 1	Patient 2
Age	5-year-old	6-year-old
Sex	Male	Male
Point of maximal tenderness	Right lower quadrant	Right lower quadrant
WBC	8.8 10^3^/ul	14.0 10^3^/ul
% Neutrophil count	81.0%	73.3%
Referral ultrasound result	Suspicious for acute appendicitis with a 0.6 cm noncompressible tubular structure in the right lower quadrant	The appendix was not visualized
Repeat ultrasound results	Possible early acute appendicitis given the appendix measures at the upper limits of normal (7 mm) with a small amount of surrounding inflammation and a trace amount of free fluid	Visualization of the appendix with borderline abnormal diameter (6-7 mm) towards the tip.
Surgical pathology results	Negative	Negative

Independent Review: A pediatric radiologist independently re-evaluated all ultrasounds. The second review did not differ meaningfully from the original reports. Pediatric ED ultrasounds tended to be more comprehensive, lasting longer on average (14.8 vs. 6.2 minutes, *p* = 0.0001) and including more cine clips and still images, consistent with a more detailed pediatric imaging approach.

## Discussion

This study demonstrates that ultrasound performed at a pediatric ED is more accurate than ultrasound performed at outside EDs. The differences likely reflect operator and interpreter expertise, as well as the availability of pediatric-specific imaging protocols (13, 14).

Importantly, the study highlights a practical pathway: when an outside ED ultrasound is nondiagnostic or equivocal, the preferred next step is transfer to a pediatric center before obtaining a CT. At the receiving center, repeating ultrasound should be planned and expected, as repeat US increases visualization and diagnostic confidence while avoiding unnecessary radiation (18–20).

Our findings are consistent with prior literature, which demonstrates that specialized pediatric radiology teams achieve higher visualization rates and lower nondiagnostic scan rates compared to community practice (13–15). This reinforces the importance of pediatric expertise rather than broader expansion of resources at small hospitals, which may not be feasible.

Furthermore, the blinded re-review of referral ultrasounds by a pediatric radiologist demonstrated that discrepancies were not due to interpretation alone but likely related to sonographer technique and study quality at the outside ED. This underscores the operator-dependent nature of pediatric appendiceal ultrasound (21, 22).

## Limitations

This study has several limitations. The retrospective design inherently carries risk of selection bias and incomplete data (23). The sample size was modest, limiting generalizability.

We only included children who had both an outside and pediatric ED ultrasound; children who had an ultrasound only at the outside ED were not captured. Studying this group in future work would provide important information.

A detailed subset analysis of patients with divergent interpretations was beyond the scope of this study. Although we reviewed basic demographics and found no obvious differences, outcomes and other subgroup findings could not be reliably determined within this retrospective design. Additionally, the time elapsed between the initial and repeat ultrasound may have influenced accuracy, as appendicitis is a progressive disease (24).

## Conclusion

Ultrasound performed at a pediatric ED demonstrated substantially higher sensitivity and specificity for appendicitis than ultrasounds performed at referring EDs. When referral ED ultrasounds are nondiagnostic or equivocal, transfer before CT and repeat ultrasound at the pediatric ED improves diagnostic accuracy and helps minimize unnecessary radiation exposure.

## Data Availability

The raw data supporting the conclusions of this article will be made available by the authors, without undue reservation.
